# (*E*)-2-(2-Methyl­cyclo­hexyl­idene)hydrazinecarbothio­amide

**DOI:** 10.1107/S1600536811042486

**Published:** 2011-10-22

**Authors:** Justin W. Hicks, Alan J. Lough, Alan A. Wilson, Neil Vasdev

**Affiliations:** a PET Centre, Centre for Addiction and Mental Health, and Institute of Medical Science, University of Toronto, 250 College Street, Toronto, Ontario, Canada M5T 1R8; b Department of Chemistry, University of Toronto, 80 St George Street, Toronto, Ontario, Canada M5S 3H6

## Abstract

In the crystal of the title compound, C_8_H_15_N_3_S, mol­ecules are linked by N—H⋯S hydrogen bonds, forming chains along [1
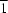
0]. An intra­molecular N—H⋯N hydrogen bond is also present.

## Related literature

The title compound, C_8_H_15_N_3_S, is a key inter­mediate for the preparation of hydrazinyl-5-aryl­thia­zole-based monoamine oxidase B (MAO-B) inhibitors. For the synthesis of hydrazinyl-5-aryl­thia­zoles and their MAO-B inhibitory activity, see: Chimenti *et al.* (2008 ▶, 2010 ▶). For background on our inter­est in radiolabelled mol­ecules targeting MAO-B, see: Vasdev *et al.* (2011*a* ▶,*b* ▶). For the preparation of ^18^F-labelled potassium cryptand fluoride, see: Vasdev *et al.* (2009 ▶).
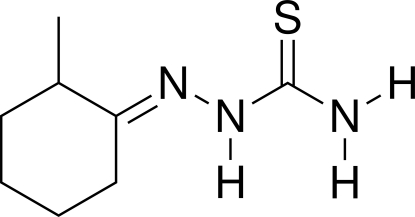


## Experimental

### 

#### Crystal data

C_8_H_15_N_3_S
*M* *_r_* = 185.29
Triclinic, 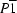

*a* = 6.0261 (5) Å
*b* = 8.0655 (4) Å
*c* = 10.9129 (9) Å
α = 83.904 (5)°
β = 89.386 (4)°
γ = 68.416 (4)°
*V* = 490.19 (6) Å^3^
*Z* = 2
Mo *K*α radiation
μ = 0.28 mm^−1^
*T* = 150 K
0.20 × 0.14 × 0.04 mm

#### Data collection

Nonius KappaCCD diffractometer
Absorption correction: multi-scan (*SORTAV*; Blessing, 1995 ▶) *T*_min_ = 0.710, *T*_max_ = 1.060
5938 measured reflections
2184 independent reflections
1698 reflections with *I* > 2σ(*I*)
*R*_int_ = 0.077

#### Refinement

*R*[*F* ^2^ > 2σ(*F* ^2^)] = 0.048
*wR*(*F* ^2^) = 0.122
*S* = 1.05
2184 reflections
122 parameters
H atoms treated by a mixture of independent and constrained refinement
Δρ_max_ = 0.26 e Å^−3^
Δρ_min_ = −0.25 e Å^−3^

### 

Data collection: *COLLECT* (Nonius, 2002 ▶); cell refinement: *DENZO-SMN* (Otwinowski & Minor, 1997 ▶); data reduction: *DENZO-SMN*; program(s) used to solve structure: *SIR92* (Altomare *et al.*, 1994 ▶); program(s) used to refine structure: *SHELXTL* (Sheldrick, 2008 ▶); molecular graphics: *PLATON* (Spek, 2009 ▶); software used to prepare material for publication: *SHELXTL*.

## Supplementary Material

Crystal structure: contains datablock(s) I, global. DOI: 10.1107/S1600536811042486/pv2460sup1.cif

Structure factors: contains datablock(s) I. DOI: 10.1107/S1600536811042486/pv2460Isup2.hkl

Supplementary material file. DOI: 10.1107/S1600536811042486/pv2460Isup3.cml

Additional supplementary materials:  crystallographic information; 3D view; checkCIF report

## Figures and Tables

**Table 1:** Hydrogen-bond geometry (Å, °)

*D*—H⋯*A*	*D*—H	H⋯*A*	*D*⋯*A*	*D*—H⋯*A*
N2—H1*N*⋯S1^i^	0.88 (3)	2.61 (3)	3.4645 (19)	162 (2)
N3—H3*N*⋯S1^ii^	0.88 (2)	2.52 (2)	3.3954 (19)	170.9 (19)
N3—H2*N*⋯N1	0.81 (3)	2.28 (2)	2.601 (2)	104.6 (19)

Symmetry codes: (i) 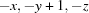; (ii) 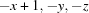.

**Fig. 1.**
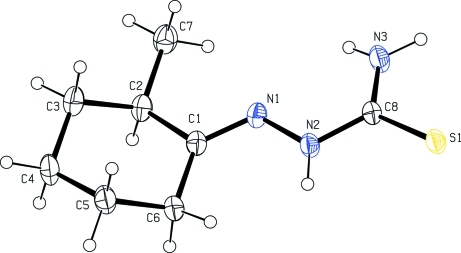
The molecular structure with ellipsoids drawn at the 30% probabilty level.

**Fig. 2.**
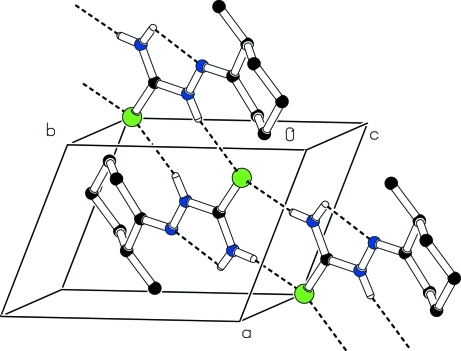
Part of the crystal structure with hydrogen bonds drawn as dashed lines. Only H atoms involved in hydrogen bonds are shown.

## supplementary crystallographic information

### Comment

(*E*)-2-(2-Methylcyclohexylidene)hydrazinecarbothioamide is an intermediate towards the preparation of hydrazinyl-5-arylthiazoles which are curerntly under exploration as a new class of inhibitors of the enzyme monoamine oxidase B (Chimenti *et al.* 2010). Our interest in this class of compounds is to prepare a radiotracer for imaging MAO-B in the central nervous system with positron emission tomography (PET). Chimenti *et al.* (2010) reported the synthesis of (*E*)-2-(2-(2-methylcyclohexylidene)hydrazinyl)-5-(4-nitrophenyl)thiazole, and (*E*)-2-(2-(2-methylcyclohexylidene)hydrazinyl)-5-(4-fluorophenyl)thiazole which demonstrated high affinity for MAO-B (K_i_ > 10 nM). We have attempted to use the 4-nitrophenyl thiazole derivative as a precursor for radiofluorination with the positron emitting isotope fluorine-18 (t_1/2_ = 109.7 min) to prepare [^18^F]-(*E*)-2-(2-(2- methylcyclohexylidene)hydrazinyl)-5-(4-fluorophenyl)thiazole. Although initial attempts to achieve this goal have not been successful due to degradation of the precursor under basic conditions, we continue to investigate the application of thiazoles as an activating group for aromatic radiofluorination.

The molecular structure of the title compound is shown in Fig. 1. In the crystal, molecules are linked by N—H···S hydrogen bonds to form chains along [110] (see Fig. 2). An intramolecular N—H···N hydrogen bond is also present.

### Experimental

*Synthesis*

The title compound, C_8_H_15_N_3_S, was obtained by stirring equimolar amounts (10 mmol) of racemic 2-methylcyclohexanone and thiosemicarbazide with a catalytic amount of acetic acid (*ca* 350 µ*L*) in 2-propanol (100 ml) for 16 h at room temperature. A white precipitate resulted and was collected by vacuum filtration and washed with cold 2-propanol (3 *x* 20 ml). This solid was then dissolved in chloroform (20 ml) and the insoluble unreacted thiosemicarbazide was removed by vacuum filtration. The solvent was removed from the filtrate by rotary evaporation and C_8_H_15_N_3_S was obtained as a white solid in 98% yield. X-ray quality crystals were obtained by slow evaporation of a solution of the title compound in 1:1:2 chloroform/acetonitrile/acetone. m.p. = 420 - 421 K.

*Attempted Radiosynthesis*

Dry ^18^F-labeled potassium cryptand fluoride ([K_222_][^18^F]; 760 µCi) was prepared as previously described (Vasdev *et al.*, 2009). A solution of 2-(2-cyclohexylidenehydrazinyl)-4-(4-nitrophenyl)thiazole in anhydrous CH_3_CN (9.5 m*M*, 1 ml) was added to the glass test tube and the solution turned a dark purple. The reaction was stirred at room temperature for 10 minutes, then an aliquot was quenched in HPLC buffer to monitor the progress of the reaction by analytical HPLC. As no reaction occurred, the mixture was then heated to 333 K and 363 K in an oil bath for 10 minutes, respectively, with still no reaction occurring. Analytical HPLC was performed using a perfluorophenyl column (Thermo Scientific Fluophase PFP, 150 *x* 10 mm, 5 µm) eluted with 70:30 CH_3_OH:H_2_O + 0.1 N ammonium formate using a flow of 5 ml min^-1^. Authentic 2-(2-cyclohexylidenehydrazinyl)-4-(4-fluorophenyl)thiazole (*t*_R_ = 12.5 min) was used as a standard.

A second reaction under microwave heating (60 W) was also attempted using dimethylsulfoxide (DMSO) as the solvent. The reaction again turned dark purple with the addition of the precursor as a DMSO solution (9.5 m*M*, 1 ml) to the dry [K_222_][^18^F] containing glass test tube. After heating to 393 K for 5 minutes with no reaction occurring, the temperature was increased to 453 K for 15 minutes. At this point, there was no precursor remaining intact, as determined by analytical HPLC. Proton NMR spectroscopy revealed that the hydrazinic proton is removed under basic conditions.

### Refinement

H atoms bonded to C atoms were placed in calculated positions with C—H = 0.98 - 1.00Å and were included in the refinement with *U*_iso_(H) = 1.2*U*_eq_(C) or 1.5*U*_eq_(C_methyl_). H atoms bonded to N atoms were refined independently with isotropic displacement parameters.

### Crystal data

**Table:**

C_8_H_15_N_3_S	*Z* = 2
*M**_r_* = 185.29	*F*(000) = 200
Triclinic, *P*1	*D*_x_ = 1.255 Mg m^−^^3^
Hall symbol: -P 1	Mo *K*α radiation, λ = 0.71073 Å
*a* = 6.0261 (5) Å	Cell parameters from 5938 reflections
*b* = 8.0655 (4) Å	θ = 2.6–27.5°
*c* = 10.9129 (9) Å	µ = 0.28 mm^−^^1^
α = 83.904 (5)°	*T* = 150 K
β = 89.386 (4)°	Plate, colourless
γ = 68.416 (4)°	0.20 × 0.14 × 0.04 mm
*V* = 490.19 (6) Å^3^	

### Data collection

**Table:**

Nonius KappaCCD diffractometer	2184 independent reflections
Radiation source: fine-focus sealed tube	1698 reflections with *I* > 2σ(*I*)
graphite	*R*_int_ = 0.077
Detector resolution: 9 pixels mm^-1^	θ_max_ = 27.6°, θ_min_ = 2.7°
φ scans and ω scans with κ offsets	*h* = −7→7
Absorption correction: multi-scan (*SORTAV*; Blessing, 1995)	*k* = −10→10
*T*_min_ = 0.710, *T*_max_ = 1.060	*l* = −13→14
5938 measured reflections	

### Refinement

**Table:**

Refinement on *F*^2^	Primary atom site location: structure-invariant direct methods
Least-squares matrix: full	Secondary atom site location: difference Fourier map
*R*[*F*^2^ > 2σ(*F*^2^)] = 0.048	Hydrogen site location: inferred from neighbouring sites
*wR*(*F*^2^) = 0.122	H atoms treated by a mixture of independent and constrained refinement
*S* = 1.05	*w* = 1/[σ^2^(*F*_o_^2^) + (0.0454*P*)^2^ + 0.1232*P*] where *P* = (*F*_o_^2^ + 2*F*_c_^2^)/3
2184 reflections	(Δ/σ)_max_ < 0.001
122 parameters	Δρ_max_ = 0.26 e Å^−^^3^
0 restraints	Δρ_min_ = −0.25 e Å^−^^3^

### Special details

**Table:**

Experimental. ^1^H NMR (CDCl_3_, 400 MHz) δ p.p.m. 8.83 (br s, 1H), 7.25 (br s, 1H), 6.48 (br s, 1H), 2.66 (m, 1H), 2.29 - 2.40 (m, 1H), 1.84 - 2.00 (m, 3H), 1.75 - 1.83 (m, 1H), 1.41 - 1.66 (m, 2H), 1.23 - 1.36 (m, 1H), 1.10 (d, *J*= 6.6 Hz, 3H). HRMS (ESI) m/*z* calcd for C_8_H_16_N_3_S, 186.1059; found 186.1064 (*M*^+^+H).
Geometry. All e.s.d.'s (except the e.s.d. in the dihedral angle between two l.s. planes) are estimated using the full covariance matrix. The cell e.s.d.'s are taken into account individually in the estimation of e.s.d.'s in distances, angles and torsion angles; correlations between e.s.d.'s in cell parameters are only used when they are defined by crystal symmetry. An approximate (isotropic) treatment of cell e.s.d.'s is used for estimating e.s.d.'s involving l.s. planes.
Refinement. Refinement of *F*^2^ against ALL reflections. The weighted *R*-factor *wR* and goodness of fit *S* are based on *F*^2^, conventional *R*-factors *R* are based on *F*, with *F* set to zero for negative *F*^2^. The threshold expression of *F*^2^ > σ(*F*^2^) is used only for calculating *R*-factors(gt) *etc*. and is not relevant to the choice of reflections for refinement. *R*-factors based on *F*^2^ are statistically about twice as large as those based on *F*, and *R*- factors based on ALL data will be even larger.

### Fractional atomic coordinates and isotropic or equivalent isotropic displacement parameters (Å^2^)

**Table:**

	*x*	*y*	*z*	*U*_iso_*/*U*_eq_	
S1	0.17188 (9)	0.22748 (6)	0.01937 (5)	0.03606 (19)	
N1	0.5032 (3)	0.4731 (2)	0.19337 (16)	0.0335 (4)	
H1N	0.185 (5)	0.522 (3)	0.104 (2)	0.051 (7)*	
N2	0.3306 (3)	0.4439 (2)	0.12560 (17)	0.0336 (4)	
N3	0.6066 (3)	0.1654 (2)	0.10779 (19)	0.0428 (5)	
H2N	0.708 (4)	0.195 (3)	0.137 (2)	0.047 (7)*	
H3N	0.649 (4)	0.061 (3)	0.078 (2)	0.039 (6)*	
C1	0.4559 (4)	0.6253 (2)	0.23460 (19)	0.0334 (5)	
C2	0.6541 (4)	0.6475 (3)	0.3062 (2)	0.0370 (5)	
H2A	0.7074	0.7344	0.2537	0.044*	
C3	0.5603 (4)	0.7327 (3)	0.4246 (2)	0.0437 (5)	
H3A	0.5190	0.6458	0.4822	0.052*	
H3B	0.6883	0.7593	0.4646	0.052*	
C4	0.3419 (4)	0.9048 (3)	0.4007 (2)	0.0476 (6)	
H4A	0.2849	0.9524	0.4799	0.057*	
H4B	0.3853	0.9959	0.3487	0.057*	
C5	0.1440 (4)	0.8698 (3)	0.3362 (2)	0.0456 (6)	
H5A	0.0066	0.9842	0.3177	0.055*	
H5B	0.0899	0.7880	0.3919	0.055*	
C6	0.2269 (4)	0.7867 (3)	0.2168 (2)	0.0407 (5)	
H6A	0.1009	0.7509	0.1837	0.049*	
H6B	0.2504	0.8778	0.1553	0.049*	
C7	0.8715 (4)	0.4756 (3)	0.3314 (2)	0.0450 (6)	
H7A	0.9305	0.4286	0.2532	0.067*	
H7B	0.8275	0.3866	0.3837	0.067*	
H7C	0.9968	0.5004	0.3735	0.067*	
C8	0.3858 (3)	0.2802 (2)	0.08743 (18)	0.0311 (4)	

### Atomic displacement parameters (Å^2^)

**Table:**

	*U*^11^	*U*^22^	*U*^33^	*U*^12^	*U*^13^	*U*^23^
S1	0.0358 (3)	0.0288 (3)	0.0448 (4)	−0.0112 (2)	−0.0042 (2)	−0.0115 (2)
N1	0.0343 (9)	0.0373 (9)	0.0338 (10)	−0.0170 (7)	0.0000 (8)	−0.0105 (7)
N2	0.0311 (9)	0.0303 (8)	0.0405 (11)	−0.0104 (7)	−0.0030 (8)	−0.0120 (7)
N3	0.0352 (10)	0.0337 (9)	0.0588 (14)	−0.0073 (8)	−0.0075 (9)	−0.0202 (9)
C1	0.0381 (11)	0.0348 (10)	0.0306 (11)	−0.0162 (9)	0.0041 (9)	−0.0089 (8)
C2	0.0385 (12)	0.0423 (11)	0.0371 (12)	−0.0210 (9)	0.0026 (10)	−0.0116 (9)
C3	0.0464 (13)	0.0503 (12)	0.0424 (14)	−0.0240 (10)	0.0000 (11)	−0.0173 (10)
C4	0.0531 (14)	0.0452 (12)	0.0505 (15)	−0.0199 (11)	0.0035 (12)	−0.0254 (11)
C5	0.0446 (13)	0.0413 (11)	0.0513 (15)	−0.0126 (10)	0.0001 (11)	−0.0190 (10)
C6	0.0478 (13)	0.0330 (10)	0.0422 (13)	−0.0137 (9)	−0.0056 (10)	−0.0116 (9)
C7	0.0378 (12)	0.0528 (13)	0.0481 (14)	−0.0185 (10)	0.0013 (10)	−0.0155 (11)
C8	0.0344 (11)	0.0291 (9)	0.0309 (11)	−0.0115 (8)	0.0031 (9)	−0.0091 (8)

### Geometric parameters (Å, °)

**Table:**

S1—C8	1.698 (2)	C3—H3A	0.9900
N1—C1	1.284 (2)	C3—H3B	0.9900
N1—N2	1.385 (2)	C4—C5	1.518 (3)
N2—C8	1.348 (2)	C4—H4A	0.9900
N2—H1N	0.88 (3)	C4—H4B	0.9900
N3—C8	1.317 (3)	C5—C6	1.523 (3)
N3—H2N	0.81 (3)	C5—H5A	0.9900
N3—H3N	0.88 (2)	C5—H5B	0.9900
C1—C6	1.506 (3)	C6—H6A	0.9900
C1—C2	1.508 (3)	C6—H6B	0.9900
C2—C7	1.518 (3)	C7—H7A	0.9800
C2—C3	1.532 (3)	C7—H7B	0.9800
C2—H2A	1.0000	C7—H7C	0.9800
C3—C4	1.521 (3)		
			
C1—N1—N2	119.69 (16)	C5—C4—H4B	109.6
C8—N2—N1	117.61 (16)	C3—C4—H4B	109.6
C8—N2—H1N	115.7 (16)	H4A—C4—H4B	108.1
N1—N2—H1N	126.7 (16)	C4—C5—C6	111.68 (19)
C8—N3—H2N	121.1 (17)	C4—C5—H5A	109.3
C8—N3—H3N	118.9 (15)	C6—C5—H5A	109.3
H2N—N3—H3N	119 (2)	C4—C5—H5B	109.3
N1—C1—C6	127.45 (18)	C6—C5—H5B	109.3
N1—C1—C2	116.52 (17)	H5A—C5—H5B	107.9
C6—C1—C2	116.01 (16)	C1—C6—C5	112.49 (18)
C1—C2—C7	113.51 (16)	C1—C6—H6A	109.1
C1—C2—C3	110.67 (17)	C5—C6—H6A	109.1
C7—C2—C3	112.02 (19)	C1—C6—H6B	109.1
C1—C2—H2A	106.7	C5—C6—H6B	109.1
C7—C2—H2A	106.7	H6A—C6—H6B	107.8
C3—C2—H2A	106.7	C2—C7—H7A	109.5
C4—C3—C2	112.57 (19)	C2—C7—H7B	109.5
C4—C3—H3A	109.1	H7A—C7—H7B	109.5
C2—C3—H3A	109.1	C2—C7—H7C	109.5
C4—C3—H3B	109.1	H7A—C7—H7C	109.5
C2—C3—H3B	109.1	H7B—C7—H7C	109.5
H3A—C3—H3B	107.8	N3—C8—N2	117.45 (18)
C5—C4—C3	110.46 (16)	N3—C8—S1	122.61 (15)
C5—C4—H4A	109.6	N2—C8—S1	119.92 (15)
C3—C4—H4A	109.6		

### Hydrogen-bond geometry (Å, °)

**Table:**

*D*—H···*A*	*D*—H	H···*A*	*D*···*A*	*D*—H···*A*
N2—H1N···S1^i^	0.88 (3)	2.61 (3)	3.4645 (19)	162 (2)
N3—H3N···S1^ii^	0.88 (2)	2.52 (2)	3.3954 (19)	170.9 (19)
N3—H2N···N1	0.81 (3)	2.28 (2)	2.601 (2)	104.6 (19)

Symmetry codes: (i) −*x*, −*y*+1, −*z*; (ii) −*x*+1, −*y*, −*z*.
